# Regulatory Pathway for Licensing Biotherapeutics in Mexico

**DOI:** 10.3389/fmed.2018.00272

**Published:** 2018-09-25

**Authors:** Carlos A. López-Morales, Alejandra Tenorio-Calvo, Rodolfo Cruz-Rodríguez, Julio Sánchez y Tepoz, Lahouari Belgharbi, Sonia Mayra Pérez-Tapia, Emilio Medina-Rivero

**Affiliations:** ^1^Unidad de Desarrollo e Investigación en Bioprocesos, Escuela Nacional de Ciencias Biológicas, Instituto Politécnico Nacional, Ciudad de Mexico, Mexico; ^2^Colegio Nacional de Químicos Farmacéuticos Biólogos México AC, Ciudad de Mexico, Mexico; ^3^Comisión Federal para la Protección contra Riesgos Sanitarios, Ciudad de Mexico, Mexico

**Keywords:** biotherapeutic products, harmonization, Common Technical Document, regulation, registration, licensing

## Abstract

Biotherapeutic products which are derived from living organisms using recombinant DNA technology significantly contribute to the progress in the treatment of life-threatening and chronic diseases. The worldwide sale of biological drugs in 2016 was near US $263,700 million. In Latin America, where monoclonal antibodies market was worth US $7000 million, being Mexico the second largest market. Approval is one of the key aspects which influences the market of medicinal products, thus it is responsibility of the regulatory authority to establish a regulatory framework that ensure safety and efficacy of the products, and it is responsibility of the applicants to provide a high quality dossier in accordance with the registration requirements of the country. The applicants submitting registration requests in Mexico need to be aware of the requirements. Similar to many other countries, Mexico has adopted the Common Technical Document (CTD) structure for organizing dossier of the medicinal product for submission into main modules (i.e., quality, non-clinical, and clinical). This facilitates the submission process of medicinal products following a logical sequence aligned to the International Council on Harmonisation (ICH) guidelines. Moreover, this structure improves the transparency and clarity of the dossier in process of evaluation of medicinal products. In Mexico, the Ministry of Health has published a regulation, NOM-257-SSA1-2014, which established the general requirements to be followed by applicants to complete the registration of biotherapeutics. This regulation stipulates that the evaluation process is supported by a regulatory framework involving Good Manufacturing Practices, labeling, stability, clinical trials, biocomparability studies, pharmacovigilance, and a technical evaluation performed by a multidisciplinary team of experts in biotherapeutics development. Additionally, the Mexican regulatory agency, COFEPRIS, has published specific guidelines to facilitate the application process. Despite the availability of this information, the scope is limited to regulatory and administrative purposes, rather than technical-scientific supporting knowledge. The aim of this article is to provide concise information to improve and promote communication between industry and regulatory agencies. Herein, we describe the current process of COFEPRIS in regulating biotherapeutics in Mexico. This process explains the basis for the organization and structure of the technical-scientific information of biotherapeutics required for registration application.

## Introduction

Biotherapeutics are obtained from living organisms by recombinant DNA technology, and they have contributed to the successful advancement of the treatment of many life-threatening and chronic diseases (1). Biotherapeutic products are larger and more complex than chemical-based drugs in their molecular structure and composition and an exhaustive characterization and development of the appropriate analytical methodologies is necessary (2). Therefore, specific regulations and robust scientific evaluations should assure the safety, efficacy, and quality throughout all the stages of the product's lifecycle.

Worldwide, the sale of biological drugs in 2016 was USD 263,700 million, and this segment increased at a compound annual growth rate (CAGR) of 3.7% (3). Products from the biotechnology segment are mainly targeted at oncological conditions, Alzheimer's disease, cardiovascular disease, diabetes, multiple sclerosis, and arthritis (3). North America is the largest market (up to 45%), followed by other developed countries of Europe and Asia. In Latin America, the monoclonal antibodies market was worth ~USD 7000 million in 2016 and is estimated to grow at a CAGR of 3.89% (4). In Latin America (LA), Mexico is the second largest market after Brazil (5). This growing market is also supported by specific regulatory frameworks, for instance the US market is supported by the Food and Drug Administration (FDA), which has established expedited programs for approval of drugs, especially biologics because of their applicability to unmet health needs (6).

In this regard, several regulatory agencies worldwide have adopted a pathway for approval that includes the evaluation of biotherapeutic products at the following three levels: (1) quality (Good Manufacturing Practices [GMPs]), (2) non-clinical studies, and (3) clinical trials (7–9). According to the information on the official site of ICH about History, during the 1980s, the European Communities (EC) advanced toward the development of a single economic market for pharmaceuticals, which led to the harmonization of the regulation of medicinal products to facilitate trade among these countries. Some years later, Japan and the US converged to harmonize their markets and joined the European Union (EU) initiative in 1997 to form the International Council on Harmonisation (ICH). ICH was developed in meetings, which initiated the formal adoption of the internationally harmonized guidelines on quality, safety, efficacy, and multidisciplinary topics (10). In 2015, the ICH changed its name to the International Conference for Harmonisation of Technical Requirements for Registration of Pharmaceuticals for Human Use.

One of the most significant contributions of the ICH in its early stage was the harmonization of technical information to be submitted for review, which established the concept of Common Technical Document (CTD). The CTD organizes the information into the following five modules: (1) administrative information (regional administrative not part of the CTD); (2) summaries of all modules, (for Mexico, the dossier should include these in the original language with their and translations in Spanish); (3) quality (in extent); (4) non-clinical study reports and; (5) clinical study reports. In 2003, the CTD format became mandatory for new drug applications in Europe and Japan and is strongly recommended for submissions to the FDA in the US (11). However, other non-ICH member countries have adopted the CTD requirements, in full or part, for their application submission. While, the WHO has not issued recommendations to adopt the CTD, it stipulates that whenever possible, the concept and adaptability to the respective country are needed as Good Regulatory Practices (GRP). WHO in its submission of the vaccine prequalification recognized the CTD and accepted it as part of the WHO prequalification process submission (12, 13).

While the CTD format has facilitated the submission of medicinal products including biotherapeutics in ICH member countries and certain non-ICH countries, most countries do not use this concept (14). As a matter of fact, Latin American (LA) countries, have moved forward through the technical support of the World Health Organization/Pan American Health Organization/Pan American Network for Drug Regulatory Harmonization (WHO/OPS/PANDRH) to develop an abbreviated version of the Canadian Health Biologics and Genetic Therapies Directorate (BGTD) process to be adopted by the LA countries (14, 15).

Mexico is one of the pioneers of the regulation of biological drugs in LA countries (5, 16). The current Mexican regulatory framework regards these products into two major groups; (1) Biologicals; which embraces vaccines, blood products, and other that do not involve DNA manipulation, and (2) Biopharmaceuticals; which embraces products obtained from recombinant DNA technology, including biosimilars. In addition, since many biologicals and biopharmaceuticals were approved before the regulatory framework was updated, the Mexican regulatory agency COFEPRIS (acronym in Spanish of the Federal Commission for Protection against Sanitary Risks) established procedures for new registration and modification of already registered products (15).

COFEPRIS was created in 2001 as a decentralized branch of the Health Ministry (SALUD) and it has an administrative and operative autonomy for issuing regulations to register all pharmaceutical products and licensing their manufacturing (17). The vision, mission, and scope of COFEPRIS covers the regulation of production, commercialization, import, export, and advertising of drugs, medical devices, health-related technologies, health at work, and risks related to environmental factors.

Furthermore, patents of many innovator drugs, including biotherapeutics, have expired or are about to expire. This has created a wide window for marketing Biosimilars (also known as biocomparables in Mexico). As a matter of fact, development of monoclonal antibodies (mAbs) is one of the growing areas of biological drug research along with vaccines, recombinant hormones, and cytokines (18).

In this article, we describe the pathway in Mexico for organizing and submitting the relevant documents for quality, non-clinical, and clinical information of biotherapeutics, and a strategy for new drug registration knowing the requirements of the Mexican regulatory framework and the criteria driving the decision making of the competent COFEPRIS evaluation committees.

## Regulation of biotherapeutics in Mexico

In Mexico, the law, regulations, and guidelines have been updated to be consistent with the published international standards of registration of both innovators and biosimilars. Furthermore, their scope is also extended to the registration of biotherapeutics authorized prior to the approval of NOM-257-SSA1-2014 “*En materia de medicamentos biotecnológicos*” (In the matter of biotechnological medicines) issued in 2014 (16, 19).

Historically, since 2009, biotherapeutics in Mexico have been regulated by the Mexican General Law of Health (LGS, an acronym of Ley General de Salud, in Spanish) and the terms “innovator,” “reference product” and “biocomparable (biosimilar)” were defined and introduced (20). In 2012, the first regulation derived from this expedited regulation “NOM-EM-001-SSA1-2012” was issued to further clarify the definition and the scope (21). However, this expedited regulation was further revised, leading to a new regulation NOM-257-SSA1-2014 that covers additional aspects related to biotherapeutics and ensures that all other aspects included in the definition are fully addressed.

The scope of the NOM-257-SSA1-2014 (22) issued in December 2014, embraces the following:

1. Guidance for the evaluation of the technical and scientific information submitted as part of the application for registration of biotechnological drugs.
2. The criteria from which the Minister of Health is authorized to regularize the approval process of biotherapeutics.
3. The general specifications for regulating the manufacturing of biotherapeutics.
4. The procedure to authorize clinical trials using biotherapeutics.
5. The requirements to be met by biotherapeutics for their consideration as reference products into the Mexican regulatory framework.

To achieve its objective, NOM-257-SSA1-2014 included other additional regulations of the Mexican regulatory framework aimed to rule and conduct all the stages related to the life-cycle of biotherapeutics including, GMPs, labeling, stabilities, clinical trials in humans, the requirements for the authorized third party laboratories that execute interchangeability, biocomparability tests, and pharmacovigilance (Table 1) (23–26).

**Table 1:** Laws that support the regulatory framework for biotherapeutics in Mexico.

**Regulation**	**Title**	**Source**
NOM-012-SSA3-2012	Establishing criteria for execution of research projects for health in humans.	http://dof.gob.mx/nota_detalle.php?codigo=5284148&fecha=04/01/2013
NOM-059-SSA1-2013	Good manufacturing practices for drug product.	http://dof.gob.mx/nota_detalle.php?codigo=5424575&fecha=05/02/2016
NOM-072-SSA1-2012	Labeling of drug and herbal products.	http://www.dof.gob.mx/nota_detalle.php?codigo=5278341&fecha=21/11/2012
NOM-073-SSA1-2005	Drug substance and drug product stability.	http://www.salud.gob.mx/unidades/cdi/nom/073ssa105.html
NOM-164-SSA1-2013	Good manufacturing practices for drug substance.	http://www.dof.gob.mx/nota_detalle.php?codigo=5303768&fecha=25/06/2013
NOM-177-SSA1-2013	Requirements for authorized third parties that perform interchangeability tests. Requirements to conduct comparability studies. Requirements for authorized third parties, research centers or hospital institutions that perform comparability tests.	http://www.dof.gob.mx/nota_detalle.php?codigo=5314833&fecha=20/09/2013
NOM-220-SSA1-2016	Pharmacovigilance operation.	http://www.dof.gob.mx/nota_detalle.php?codigo=5490830&fecha=19/07/2017

## Center for excellence in cofepris

To address the gap in knowledge in regulatory sciences, such as the critical thinking model and the principles and criteria required for the evaluation of biotherapeutics, COFEPRIS established a Center for Excellence in December 2016. This initiative aims to invest in regulatory affairs training programs. The aim is to achieve the standards of other regulatory authorities (mainly of developed countries), including the most advanced scientific professional from industry in regulatory and pharmaceutical sciences worldwide. In addition, it aims to develop and deliver continuous staff learning and training sessions in understanding regulatory sciences and the use of translational sciences for daily work. The expectation is to increase the interaction and synergies between regulators, academia, and the industry to improve access to health, high-quality services, and the preventative effects for the benefit of the end user. COFEPRIS through its Center of Excellence also expects a continuous quality improvement of the regulatory system in Mexico and contributes through its initiatives to the WHO universal health coverage paradigm (Table 2).

**Table 2:** Aims of Center for Excellence of COFEPRIS.

Promote	Research and Development in Regulatory Sciences including. Pharmaceutical Sciences
Disseminate	Disseminate existing and new knowledge and learning in Good Regulatory Practices to close the gap.
Moving forward	COFEPRIS leadership by moving forward the harmonization, convergence and reliance agenda.
Facilitate	Support national health institutions to engage into regulatory sciences and good regulatory practices.
Catalyze	Links Academy-Industry-Government institutions to develop and support a national strategic plan on regulatory sciences that contribute nationally, regionally and globally to better health.

*https://www.gob.mx/cofepris/acciones-y-programas/centro-de-excelencia-en-ciencia-regulatoria-y-buenas-practicas-regulatorias-coe*.

## Regulatory strategy

### Experience with biotherapeutics

**Figure 1:**
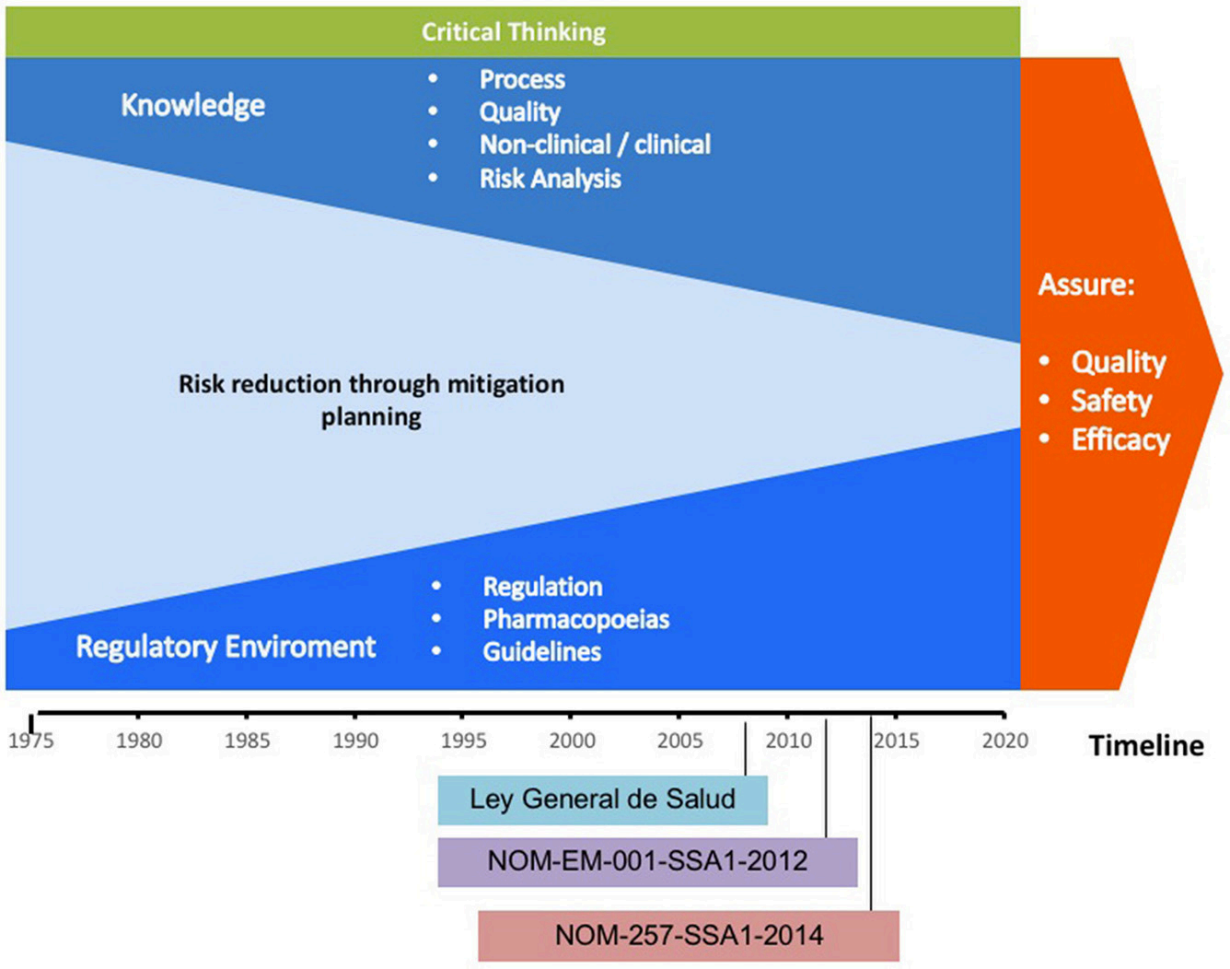
Scheme representing the gain of knowledge and the criteria for issuing regulatory decisions throughout time. Please note that as knowledge increases through the years, the risk is reduced, while critical thinking is always present.

In the last four decades, the technological improvements allowed knowledge about the design, development, production, and non-clinical/clinical assessment of new therapies for the treatment of chronic-degenerative diseases based on target-specific engineered biotherapeutics. This led to the establishment of the criteria to draft the laws, regulations, and pharmacopeias, including guidance documents for the evaluation of biotherapeutics. It has contributed to lowering the occurrence of adverse events to ensure the quality, safety, and efficacy of biotherapeutics. Figure 1 shows that the risk for issuing a regulatory decision was lowered as the cumulative scientific knowledge was raised and the evaluation criteria became more stringent from the 1970s to 2018 (27).

### Technical information submitted for evaluation

Since its creation, COFEPRIS made several efforts to align its scientific evaluation process and harmonization with ICH and WHO to achieve the standards of regulatory agencies of Europe, USA, Japan, and Canada. Accordingly, NOM-257-SSA1-2014 aimed to guide the industry to commit and comply with the assured quality standards worldwide (22). COFEPRIS, while recognized as an observer of the ICH since 2017, has for long been making efforts to harmonize with the ICH recommendations on quality, non-clinical, and clinical biotherapeutics that have to be included in the application submissions for registration.

In Mexico, a significant number of applicants were not familiar with the formal submission process of registration. Consequently, COFEPRIS issued an official guideline to facilitate the industrial applicants in their submission process. It is stated that for each biotherapeutic product, the evaluated characteristics must be described and supported by strong scientific evidence (1).

**Figure 2:**
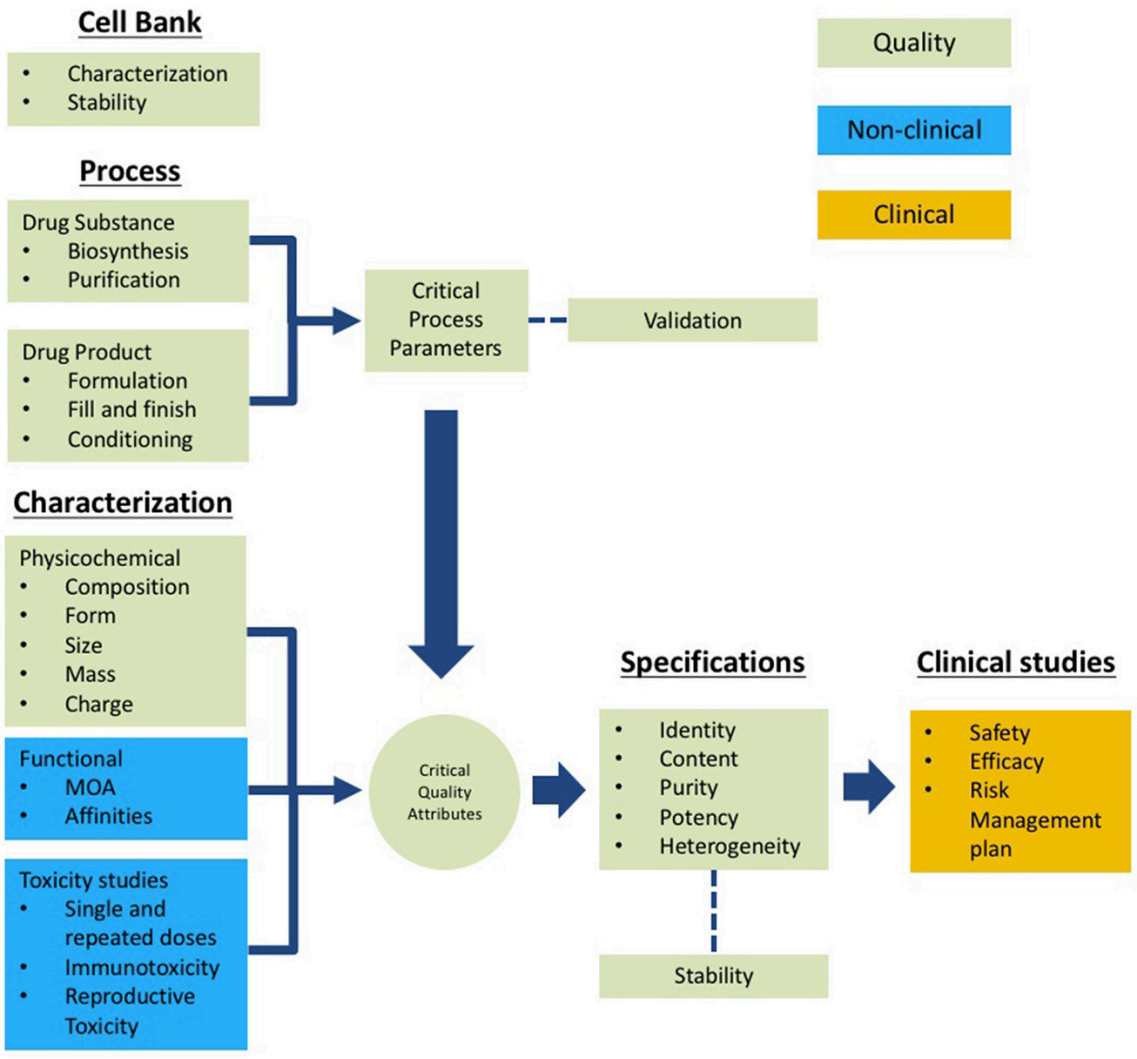
Scheme of the technical information to be submitted for registration of biotherapeutics based on the Common Technical Document (CTD) structure. Quality (green), non-clinical (blue) and clinical (orange).

The information required for the submission of biotherapeutics must follow a logical sequence and structure after an in-depth analysis that documents comply with the technical-scientific evidence that leads to the assessment of the quality, safety, and efficacy data of the product. This is the minimum level of knowledge that enhances the decision-making and encompass the critical thinking model of the concerned regulatory framework. The scheme presented in Figure 2 summarizes the structural information that should be presented to COFEPRIS regulators. This scheme includes the following components:

1. **Cell bank information:** This section should include characterization data to confirm the cell line identity, purity, and genetic stability. This component demonstrates that the cell line maintains the recombinant-gene that expresses the target protein after the number of passages established according to the quality procedures (24, 28).
2. **Process for Drug Substance (DS) and Drug Product (DP):** This section includes process mapping steps including the critical process parameters (CPPs) for each critical process step. The acceptance criteria of the process validation protocol should be based on the quality target product profiles (QTPPs) intervals of the Critical Quality Attributes (CQAs) to demonstrate the consistency of manufacturing with at least three batches documented in the corresponding validation report (24, 29–31).
3. **DS and DP characterization:** This section includes the use of orthogonal analytical methodologies and bioassays to assess the physicochemical and functional properties of the product (composition, shape, size, mass and charge, affinities, and mechanism of action). The characterization determines such properties that might impact its functionality and helps to establish its CQAs that should be considered in the quality specifications (27, 31).
4. **Specifications:** The information in this section ensures the batch-to-batch consistency of CQAs for identity, content, purity, potency, and heterogeneity. For this purpose, the use of methodologies suitable for the evaluation of such CQAs should be supported by a validation exercise. The specifications also should be addressed to demonstrate long-term stability during the shelf-life of the drug product following the same rationale of the batch consistency CQAs (24, 29, 30, 32).
5. **Non-clinical studies:** The overall goal of the non-clinical studies of biotherapeutics is to provide proof of principle for the mode of action and to identify any relevant potential toxic effects. Effector functions, tissue cross-reactivity, immunogenicity, and stability are major safety concerns for biotherapeutics. Importantly, the identification of relevant species for testing toxicity, the understanding of target antigen-antibody interaction, and the interpretation of results regarding the exposure-response relationship are critical elements underlying the design of a successful evaluation of non-clinical safety (33, 34).
6. **Clinical studies:** This section is organized to include efficacy data to support one or multiple indications, followed by the efficacy reports demonstrating primary and secondary clinical endpoints targeted at increasing the survival rate or improving the quality of life noticeable for the patient or both, or addressing disease worsening. Safety under the proposed conditions of use and reports of adverse events are included. The need to assess potential immunogenicity is relevant for biotherapeutics (35, 36). Furthermore, Mexico has improved the system for monitoring, reporting, assessing, and preventing adverse drug reactions for all pharmaceutical products. Continuous evaluation of the benefit-risk balance and the necessary regulatory action has been considered, which has improved the patient follow-up and communication with physicians. Additionally, the list of evaluated products is published on the COFEPRIS website; the physicians can be given updates about the products that have been approved.

It should be noted that the information for submission should be organized into modules comprising quality, non-clinical, and clinical studies following the CTD structure (Figure 2). The information submitted for approval of COFEPRIS should include all stages of the life cycle of the biopharmaceutical, from the origin of cell banks through the results of clinical studies. For this purpose, COFEPRIS has adopted the CTD structure.

### Registration of biosimilars (biocomparables)

This section demonstrates that the CQAs of the active substance in the biosimilar should be within the same range of variance of the reference product. In Mexico, the term to refer to Biosimilarity is Biocomparability. Demonstrating biocomparability requires comparability exercises, through an exhaustive analysis, using orthogonal techniques to evaluate physicochemical properties and functional analyses. The clinical comparability exercise is a stepwise procedure that should begin with PK and PD clinical trials that extensively evaluate the tolerability and immunogenicity of the biosimilar.

**Figure 3:**
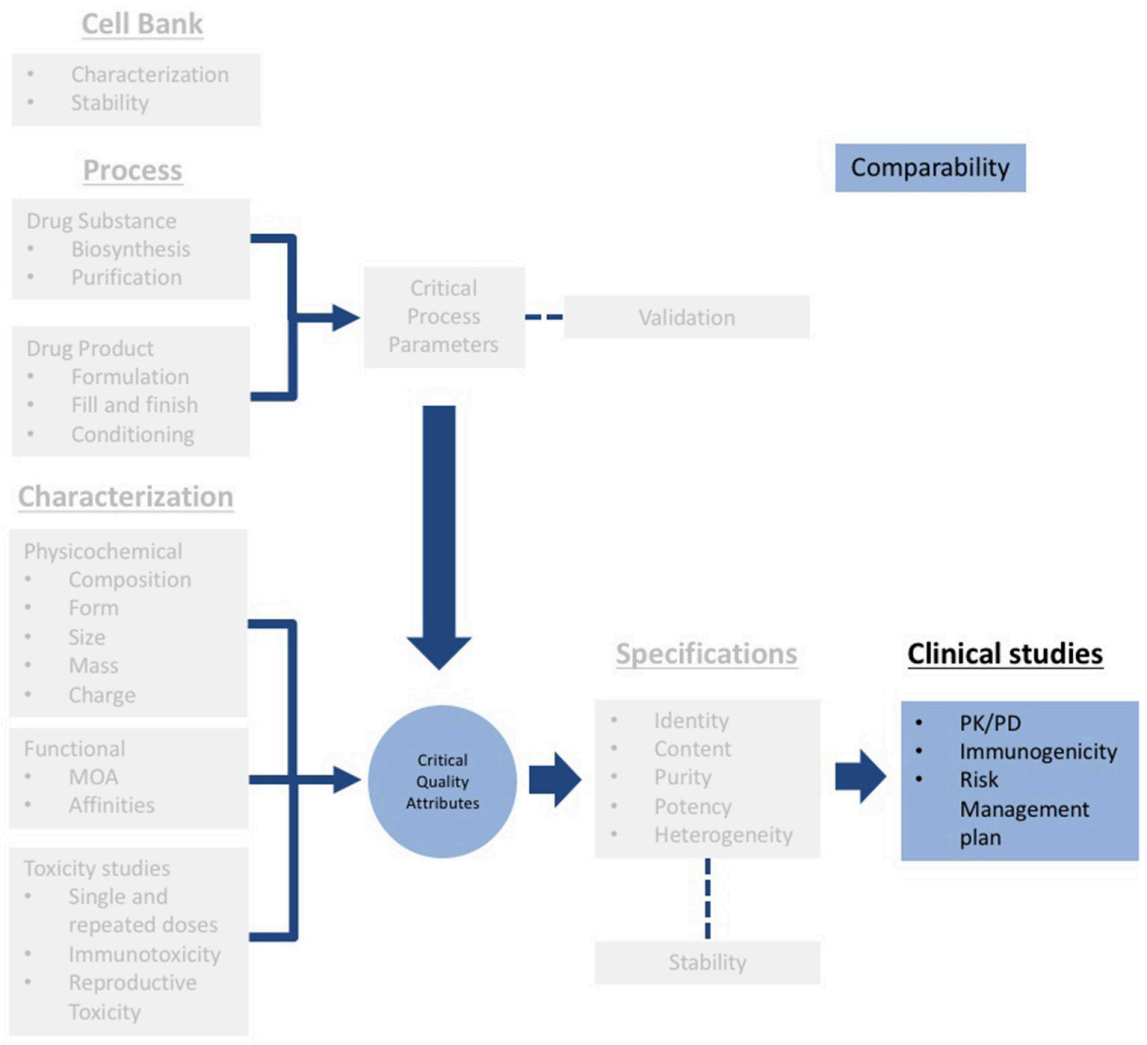
Scheme of the technical information to be submitted for registration of biosimilars (gray). Note that a specific section for biocomparability (blue) should be included, and clinical information should include PK/PD studies, immunogenicity assessment and a risk management plan.

According to the guidelines, the extent of physicochemical and functional studies will be determined by the degree of biocomparability (37, 38). In other words, the more comparable the attributes of the biosimilar concerning the attributes of the reference product are, the less non-clinical and clinical evidence will be required by the authorities. Under this rationale a highly similar molecule in terms of physicochemical and functional properties, will result in highly similar safety and efficacy profiles (Figure 3). The information required to register biosimilars has to be structured following the same scheme mentioned above as for the “reference product” (27, 37). It should be noticed that a Biocomparability section must be included in the submission.

In addition, since each biotherapeutic (cytokines, hormones, mbA, and fusion proteins) has different characteristics that need to be evaluated, COFEPRIS has released specific guidelines on the information that should be presented to demonstrate biocomparability (39). These guidelines allow the user to focus on the physicochemical and functional attributes that are relevant for each single molecule. However, in some cases, it is likely that a specific guideline has not been issued for all available biotherapeutics; thus, the user could invoke the guidelines of the molecule that have closer characteristics to those of the molecule of interest.

**Figure 4:**
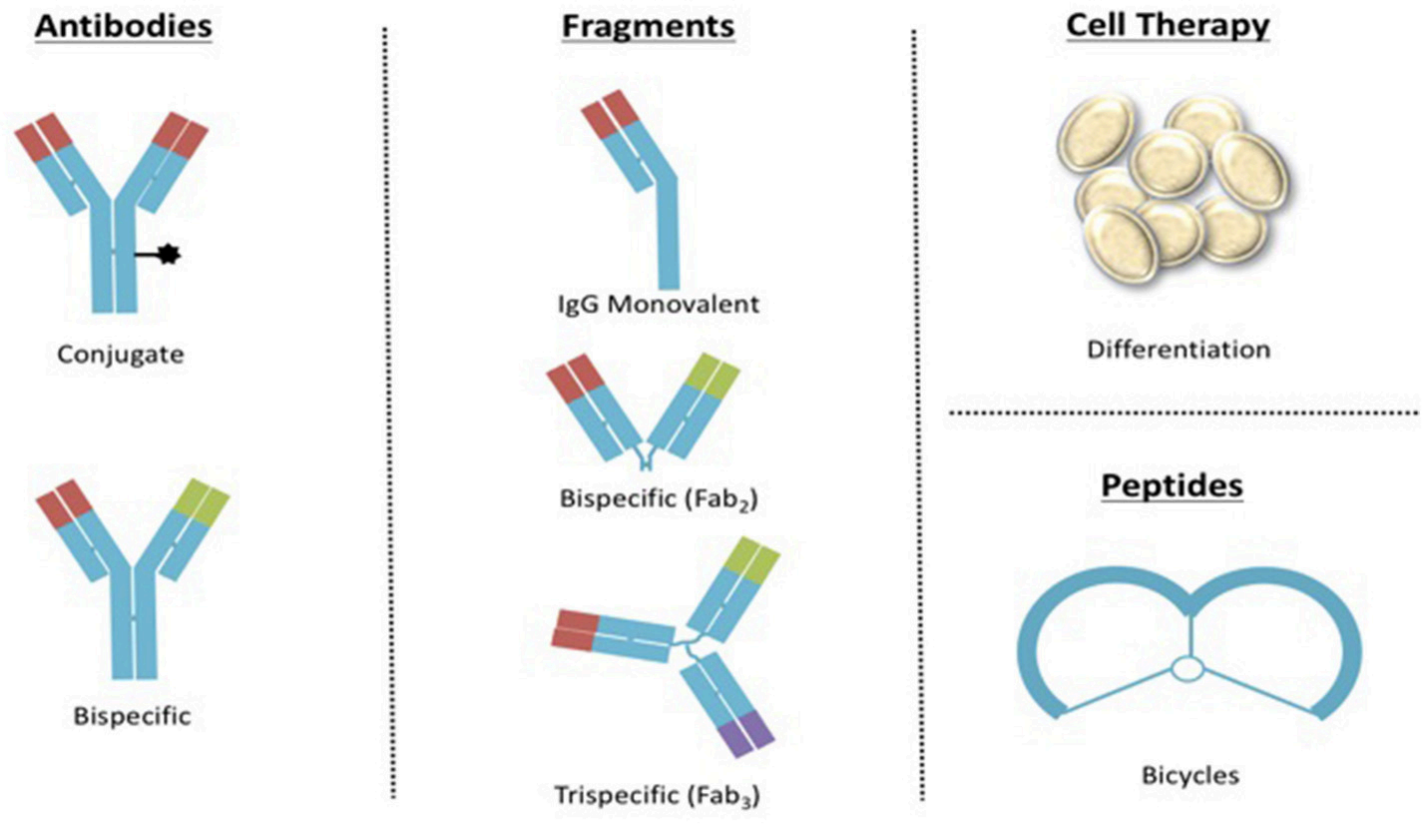
Examples of upcoming formats of biotherapeutics to be regulated in Mexico.

New formats of biotherapeutic molecules are being developing around the world. Thus, it is important to invest in regulatory science for controlling new entities based on the IgG format such as antibody-drug conjugates (ADCs) and bispecific, or IgG fragments that could include monovalent, bispecific, trispecific, and even more complex biodrugs similar to those designed for cell therapy or bicycles (Figure 4).

## Concluding remarks

COFEPRIS has been updated and implemented the current regulatory framework for the evaluation of biotherapeutics in Mexico. This evaluation process has been improved by the adoption of the CTD structure and, thus, strengthening the critical thinking of experts on regulatory authority, resulted in the approval of 19 innovator biotherapeutics and 7 biocomparables since 2015. Additional efforts are on the way to improve the knowledge and skills of regulatory experts in regulatory sciences, this will promote their critical thinking and a well-informed decision making. Accordingly, Center for Excellence of COFEPRIS have issued several training courses in collaboration with national and international institutions. In addition, international meetings have been conducted to promote networking and review of several topics in the area of regulatory sciences.

## Author contributions

All authors listed have made a substantial, direct and intellectual contribution to the work, and approved it for publication.

### Conflict of interest statement

The authors declare that the research was conducted in the absence of any commercial or financial relationships that could be construed as a potential conflict of interest.
